# The stress hormone norepinephrine increases the growth and virulence of *Aeromonas hydrophila*

**DOI:** 10.1002/mbo3.664

**Published:** 2018-06-13

**Authors:** Jinwei Gao, Bingwen Xi, Kai Chen, Rui Song, Ting Qin, Jun Xie, Liangkun Pan

**Affiliations:** ^1^ Key Laboratory of Freshwater Fisheries and Germplasm Resources Utilization Ministry of Agriculture Freshwater Fisheries Research Center Chinese Academy of Fishery Sciences Wuxi China; ^2^ Hunan Fisheries Science Institute Changsha China; ^3^ College of Fisheries and Life Science Shanghai Ocean University Shanghai China

**Keywords:** *Aeromonas hydrophila*, growth, norepinephrine, stress, virulence

## Abstract

Stress is an important contributing factor in the outbreak of infectious fish diseases. To comprehensively understand the impact of catecholamine stress hormone norepinephrine (NE) on the pathogenicity of *Aeromonas hydrophila*, we assessed variations in bacterial growth, virulence‐related genes expression and virulence factors activity after NE addition in serum‐SAPI medium. Further, we assessed the effects of NE on *A. hydrophila* virulence in vivo by challenging fish with pathogenic strain AH196 and following with or without NE injection. The NE‐associated stimulation of *A. hydrophila* strain growth was not linear‐dose‐dependent, and only 100 μM, or higher concentrations, could stimulate growth. Real‐time PCR analyses revealed that NE notably changed 13 out of the 16 virulence‐associated genes (e.g. *ompW*,* ahp*,* aha*,* ela*,* ahyR*,* ompA*, and *fur*) expression, which were all significantly upregulated in *A. hydrophila* AH196 (*p *<* *0.01). NE could enhance the protease activity, but not affect the lipase activity, hemolysis, and motility. Further, the mortality of crucian carp challenged with *A. hydrophila* AH196 was significantly higher in the group treated with NE (*p *<* *0.01). Collectively, our results showed that NE enhanced the growth and virulence of pathogenic bacterium *A. hydrophila*.

## 1 INTRODUCTION

*Aeromonas hydrophila* is ubiquitously distributed in freshwater habitats, and a well‐known opportunistic pathogen of fish, amphibians, reptiles, and mammals (Altwegg & Geiss, 2008; Pang et al., 2015; Parker & Shaw, 2011). *A. hydrophila* frequently causes hemorrhagic septicemia disease in cultured and feral fishes, such as carp, catfish, perch, and tilapia (Handfield, Simard, Couillard, & Letarte, 1996; Hossain et al., 2014). Although *A. hydrophila* receives much notoriety as a common bacterial pathogen of cultured fish, it is also indigenous to natural ecosystem, and present in the intestine of healthy fish (Zhang, Guan, Huang, & Xiong, 2013). Stress is widely considered to be an important contributing factor in the outbreak of infectious fish diseases. Host stress hormones like cortisol and norepinephrine (NE) induce comprehensive physiological activities in fish and affect the defense capabilities of fish immune systems (Fabbri, Capuzzo, & Moon, 1998; Verburg‐Van Kemenade, Ribeiro, & Chadzinska, 2011; Weyts, Cohen, Flik, & Verburg‐Van Kemenade, 1999). Recent researches have also suggested that stress hormones can significantly influence the infectivity of pathogenic bacteria (Belay, Aviles, Vance, Fountain, & Sonnenfeld, 2003; Li et al., 2015; Lyte & Ernst, 1992; Neal et al., 2001).

The catecholamine stress hormone NE is mainly released from sympathetic nerve terminals, and maintains a highly conserved molecular structure in vertebrates including fish, amphibians, and mammals (Freestone, Haigh, & Lyte, 2007; Nakano, Takahashi, Sakai, Kawano, et al., 2007). Pioneering research by Lyte and Ernst (1992) showed that catecholamine could induce the growth of Gram‐negative bacteria like *Escherichia coli*,* Yersinia enterocolitica*, and *Pseudomonas aeruginosa* in low‐nutrient, serum‐based SAPI medium. The effects of NE on growth have since been verified in many bacterial pathogens including *Listeria monocytogenes* (Coulanges, Andre, Ziegler, Buchheit, & Vidon, 1997), *A. hydrophila* (Kinney, Austin, Morton, & Sonnenfeld, 1999), *Campylobacter jejuni* (Cogan et al., 2007), and multiple *Vibrio* species (Nakano, Takahashi, Sakai, Kawano, et al., 2007). Nevertheless, not all bacteria strains exhibited positive growth in response to NE. *Porphyromonas gingivalis* growth was not affected by NE (Belay et al., 2003), and the addition of NE limited the growth of *Prevotella intermedia* and *Eikenella corrodens* (Jentsch, Marz, & Kruger, 2013). Other than facilitating growth, NE was also found to affect the production of virulence factors in pathogens, including the motility of *Salmonella enterica* serovar Typhimurium (Bearson & Bearson, 2008), *Escherichia coli* O157:H7 (Bansal et al., 2007) and *Vibrio harveyi* (Yang, Anh, Bossier, & Defoirdt, 2014), and biofilm formation of *Staphylococcus epidermidis* (Lyte et al., 2003), *Vibrio harveyi* (Yang et al., 2014), and *Streptococcus pneumonia* (Sandrini, Alghofaili, Freestone, & Yesilkaya, 2014). Thus, host stress and stress hormones play important roles in the infectivity of opportunistic pathogenic bacteria.

In this study, we examined the effects of stress hormone NE on the growth, gene expression of selected virulence factors, lytic enzyme activity, hemolysis, and swimming motility of *A. hydrophila*. Moreover, we evaluated the impact of NE on the virulence of *A. hydrophila* in crucian carp *Carassius auratus gibelio* via in vivo challenge.

## 2 MATERIALS AND METHODS

### 2.1 Bacterial strains, culture conditions, and reagents

*Aeromonas hydrophila* strains AH33, AH189, AH196, and AH301 (Table 1) were isolated from diseased carps and identified based on *gyrB* sequences. Strain NJ‐35 was donated by Prof. Yongjie Liu (College of Veterinary Medicine, Nanjing Agricultural University, China) (Pang et al., 2015). Stock cultures were maintained at −80°C in Luria‐Bertani broth (Oxoid, Basingstoke, UK) containing 30% (v/v) glycerol (Sangon Biotech, Shanghai, China). When required, the stocks were streaked on nutrient agar, incubated at 30°C overnight, and single colonies were collected and used in subsequent experiments.

**Table 1:** *Aeromonas hydrophila* strains used in this study

Strain	Source or reference
AH33	Intestine of diseased *Megalobrama amblycephala*
AH189	Blood of diseased *Megalobrama amblycephala*
AH196	Ascites of diseased *Ctenopharyngodon idella*
AH301	Kidney of diseased *Megalobrama amblycephala*
NJ‐35	Diseased *Carassius auratus* (Pang et al., 2015)

The catecholamine hormone NE (noradrenaline bitartrate) was purchased from Target Molecule (Boston). Before each experiment, NE solutions were freshly prepared with sterilized physiological saline solution and filter‐sterilized using 0.22 μm MCE syringe filters (Sangon Biotech, Shanghai, China).

Serum‐SAPI medium was prepared as described by Lyte and Ernst (1992) and Dong et al. (2016) with slight modification. Briefly, the medium contained 0.4990 g glucose, 0.5003 g NH_4_NO_3_, 0.2504 g KH_2_PO_4_, 0.2497 g KCl, and 0.1216 g MgSO_4_ in one liter of 10 mM HEPES buffer, which was supplemented with 10% (v/v) fetal bovine serum (FBS, Zhejiang Tianhang Biotechnology, Hangzhou, China).

### 2.2 Growth assays

#### 2.2.1 Trial one

*A. hydrophila* AH196 was grown in nutrient broth (Oxoid, Hampshire, England) at 30°C for 16−18 hr. Broth cultures were pelleted by centrifugation (8,000 *g*, 5 min), washed, and resuspended in stroke‐physiological saline solution in order to achieve a diluted concentration of 10^2^ colony‐forming units (CFU)/ml. Therefore, an initial inoculum density of AH196 (~10^2^ CFU/ml), which is designed to present overall bacterial proliferation process (O'Donnell, Aviles, Lyte, & Sonnenfeld, 2006), was applied to subsequent experiments.

Serum‐SAPI medium containing 10% (v/v) FBS (pH 7.2 ± 0.2) was used to assay growth capacity. One‐hundred microliters of *A. hydrophila* AH196 was inoculated in the medium containing NE (final concentration of 0, 12.5, 25, 50, 100, and 200 μM) and then incubated at 30°C with shaking at 180 rpm. Cell concentrations (OD_600_) were detected with a Multiskan GO spectrophotometer (Thermo Scientific, Waltham) at 0, 18, 24, 36, 48, 60, and 72 hr, respectively. Tests were repeated twice and with four replicates of each concentration.

#### 2.2.2 Trial two

To confirm the effect of NE on the growth of *A. hydrophila* strains AH33, AH189, AH301, and NJ‐35, the strains were inoculated in serum‐SAPI medium with and without 100 μM NE. The turbidity at 600 nm was then measured at 36 hr. Trials were repeated twice and four replicates were conducted for each bacterial strain.

### 2.3 Analysis of gene expression by quantitative RT‐PCR

*A. hydrophila* AH196 cells were cultured in serum‐SAPI medium containing 10% FBS to exponential phase (OD_600_, 0.6) with 0 and 100 μM NE treatment, collected by centrifugation (8,000 *g*, 5 min), and washed twice with sterilized physiological saline. The pellets were resuspended with precooled RNAiso Plus (Takara, Dalian, China) and frozen at −80°C. Total RNA was then isolated following the guide of RNAiso Plus kit (Takara, Dalian, China), and RNA quantities and concentrations were measured with a Nanodrop 2000 Spectrophotometer (Thermo Scientific, Waltham). Virulence‐related gene expression analyses were performed in triplicate with qRT‐PCR using the Takara one‐step SYBR^®^ PrimeScript^™^ PLUS RT‐PCR kit (Takara, Dalian, China). The reaction solutions were prepared with 100 ng RNA as template, and the following PCR amplification protocol: 42°C for 5 min and 95°C for 10 s for the reverse transcription reaction, followed by 40 cycles of 95°C for 5 s, 58°C for 34 s and 72°C for 30 s. All samples were analyzed in triplicate and the transcription levels of target genes were normalized to the expression of the housekeeping gene *rpoB*, and then calculated with the 2^−ΔΔCT^ method. Primers were designed using the NCBI online primers design tool Primer‐Blast (https://www.ncbi.nlm.nih.gov/tools/primer-blast/) (Table 2).

**Table 2:** Primers used in this study

Gene	Primer sequences (5′ → 3′)	Description	Amplicon size (bp)
*aerA*	CACGTCCATGTCTTCACCGA AGCGCGAATTTCATCAAGCC	Toxin: aerolysin	102
*ast*	CTATGAGCTGAGCGATGGCA TCCCGTCGAACTTGAAGTGG	Toxin: heat‐stable cytotonic enterotoxin	119
*ahp*	TCTATGCGCTGGAGTCGTTC AGGACATGCCCACGTTGTAG	Enzyme: serine protease	174
*act*	TCAAGGCCGATGTCAGCTAT GTCCCACTGGTAACGAATGC	Enzyme: cytolytic enterotoxin	158
*hly*	TCTACCTCAACGTCAACCGC TCCGCACTATCTTGGCATCC	Toxin: hemolysin	189
*alt*	TGGATGCCGAGCAGAACAT CTCTTTCACCGAAGTCACGC	Toxin: heat labile cytotonic enterotoxin	149
*lip*	CACCTATACCCTGAGCGTGA GAAGTAAGGCAGCTTGACGG	Enzyme: lipase	178
*ela*	TACCGCAACTGGTACAACAC CGGAGTTCTGCTCGGTAAAG	Enzyme: elastase	196
*aha*	AAGCCGTCAAGGTTACTGAC GTCACCAGTGTTGTTGGTCT	Adhesion: adhesin	182
*sodB*	CCGAGTTTGAAGGCAAGTCT GACTTGGTGAACGCATCCTT	Oxidative stress: ferrous superoxide dismutase	205
*flaA*	AGCATCAGCTCTCAAAGTGG CACTGACGTTCTCCGAGATG	Motility and adhesion: polar flagellin A	154
*flaB*	CAGTCTGAACCAGACAGGTG CAGCCATTACGTTTTGAGCC	Motility and adhesion: polar flagellin B	170
*ompW*	TACTTCGGTGATGCCAACAG CATTGATCGCCATGTCCAGA	Porin and adhesion: outer membrane protein W	166
*ompA*	TGGATCTGCAAGCTCGTTAC CTACGTAGGAAGTGCGGAAC	Porin and adhesion: outer membrane protein A	144
*fur*	ATTGGTCTCGCTACCGTCTA CGGAGAACTCGATCACCTTG	Iron acquisition and regulation: ferric uptake regulator	163
*ahyR*	GCGGTGATGAACGACAGTAT GCAGACCTTGCCCATTTACT	Quorum system: LuxI/R‐type response regulator	168
*rpoB*	ACCGACGAAGTGGACTATCT CGGCGTTCATAAAGGTGGAT	Housekeeping gene: RNA polymerase beta subunit	145

### 2.4 Protease and hemolysis assays

*A. hydrophila* AH196 was grown to exponential phase (OD_600_ of 0.6) in serum‐SAPI media with 0 and 100 μM NE added. Broth cultures were centrifuged and the supernatants were filtered through 0.22 μm MCE membrane filters.

The protease activity of *A. hydrophila* AH196 was examined using azocasein (Sigma, St. Louis) as an enzyme substrate based on methods described in Chu, Zhou, Zhu, and Zhuang (2014). Briefly, 1 ml of azocasein (3 mg/ml in 50 mM Tris–HCl buffer, pH 7.5) was added to 150 μl of AH196 supernatant, and then incubated for 30 min at 37°C. The reaction was terminated by adding 10% precooled trichloroacetic acid (500 μl) and the supernatant was collected after centrifugation. The supernatant (100 μl) was neutralized with isopyknic 1 N NaOH in 96‐well plates, and the absorbance was then measured at 400 nm with a Multiskan GO spectrophotometer.

The hemolysis activity of AH196 was measured using 4% sheep erythrocyte (Nanjing SenBeiJia, Nanjing, China) as a substrate based on modified methods that were previously described (Luo et al., 2016). Sheep erythrocyte (4%) was centrifuged and washed with phosphate buffer (PBS, pH 7.4). Five microliters of washed erythrocyte was then incubated at 37°C with 245 μl of the culture supernatant, PBS (negative control), or 1% Triton X‐100 (positive control, 100% lysis for sheep erythrocytes), respectively. After 30‐min incubation, the reaction mixture was centrifuged (2700 *g*, 10 min), and the absorbance of the supernatant (200 μl) was measured at 540 nm using a spectrophotometer. Hemolytic activity (%) was defined as [(OD_540_ sample − OD_540_ negative control) × 100]/OD_540_ positive control. All assays were repeated twice with four replicates.

### 2.5 Lipase and motility assays

Lipase and motility assays followed methods described by Yang et al. (2014) with some modifications. *A. hydrophila* AH196 was grown in nutrient broth overnight, pelleted, washed, and diluted to 1 × 10^7^ CFU/ml. A 5 μl aliquot of bacterial suspension was spotted on the center of experimental plates. After autoclaved sterilization, two types of agar were mixed with NE (100 μM final concentration) for lipase and motility assessment. Control plate agar was mixed with equal volumes of vehicle solvent. Lipase assay plates were made by supplementing serum‐SAPI agar with 1% (v/v) Tween 80 (Sinopharm, Shanghai, China). After incubation for 48 hr at 30°C, opalescent zones and colony diameters were measured, and the ratio between both parameters was calculated to measure lipase activity. The motility assays were performed on semisolid agar plates (serum‐SAPI medium + 0.5% (wt/v) agar) and diameters of swimming motility halos were determined after incubation for 24 hr at 30°C. Both lipase and motility assays were conducted twice with four technical replicates each time.

### 2.6 Crucian carp challenge test

Juvenile crucian carp (*Carassius auratus gibelio*; 48.1 ± 2.5 g and 12.1 ± 1.1 cm) were obtained from the experimental station of the Freshwater Fisheries Research Centre at the Chinese Academy of Fishery Sciences. Prior to challenging, a total of 120 fish were acclimatized in 70 × 50 × 40 cm^3^ aquariums, at a temperature of 29.5 ± 1.0°C, dissolved oxygen >5 mg/L, and given commercial feed three times each day. Fish (*n* = 120) were divided evenly into four groups with three replicates: AH196 + NE, AH196, NE, and the control group. *A. hydrophila* AH196 was grown overnight in serum‐SAPI medium at 30°C. Broth cultures were centrifuged at 8,000 × *g* for 5 min, washed twice, and diluted to 1 × 10^6 ^CFU/ml with sterile physiological saline. Fish in the AH196 + NE and AH196 groups were intraperitoneally injected with 200 μl of *A. hydrophila* AH196 suspension, while the other groups were administered 200 μl sterile physiological saline. At 4 hr postinjection, the AH196 + NE and NE groups were intraperitoneally injected with 100 μl of NE (100 μM), while fish in the other groups were injected with 100 μl of stroke‐physiological saline solution. Fish were observed in 6 hr intervals, and dead fish were removed for traditional bacteriological inspection. The holistic survival percentage was analyzed and expressed as a Kaplan–Meier survival curve with a log‐rank test. The challenge tests were carried out under the instruction and supervision of the Ethical Committee for Animal Experiments of Nanjing Agricultural University (Nanjing, China). All animal procedures abided by the guidelines of laboratory animal welfare ethical review and regulations for the administration of affairs concerning experimental animals in China.

### 2.7 Statistical analysis

All data are presented as the mean ± *SD*. The growth assay data were analyzed by one‐way ANOVA followed by Tukey's post hoc tests. Data from the gene expression profiles, protease, hemolysis, lipase, and motility assays were analyzed by Welch's *t* test. The survival of crucian carp was analyzed and expressed as a Kaplan–Meier survival curve with a log‐rank (Mantel–Cox) test. A probability (*p*) value < 0.05 was considered as statistically significant, and a probability (*p*) value < 0.01 was considered as extremely significant. All figures were plotted using the GraphPad Prism program version 7 (https://www.graphpad.com/, RRID: SCR_002798).

## 3 RESULTS

### 3.1 Growth response of *Aeromonas hydrophila* to NE

**Figure 1:**
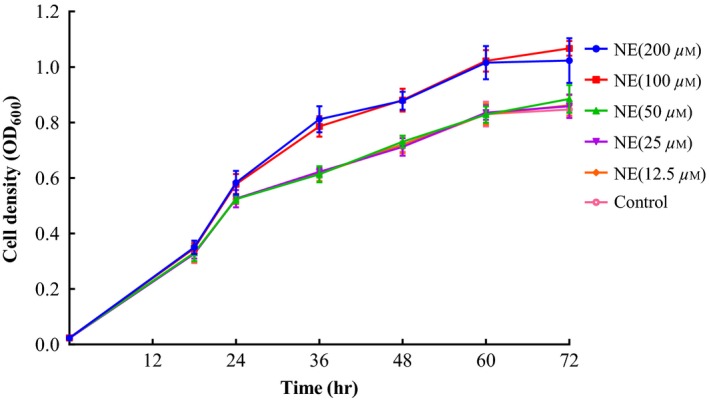
Effect of different concentrations of the catecholamine norepinephrine (NE) on the growth of *Aeromonas hydrophila* AH196 in serum‐SAPI medium supplemented with 10% fetal bovine serum. For some points, the error bars showing *SD* of eight replicates are shorter than the height of the symbol. NE (200 μM), indicates the addition of 200 μM NE; NE (100 μM), indicates the addition of 100 μM NE, and so forth; the control was supplemented with an equal dosage of sterile saline

**Figure 2:**
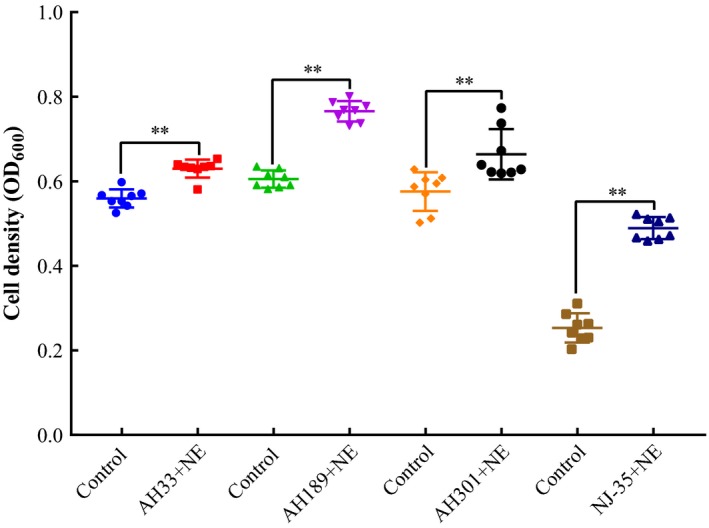
Growth of *Aeromonas hydrophila* strains that were isolated from distinct organs of cyprinid fish after exposure to norepinephrine (NE) for 36 hr in serum‐SAPI medium containing 10% fetal bovine serum. Four *Aeromonas hydrophila* strains were examined and exposed to 100 μM NE or equivalent volumes of normal saline in the experimental and control groups, respectively (**p < 0.01)

To investigate the response of *A. hydrophila* AH196 growth with NE in vitro, minimal nutrient, low‐iron SAPI medium that was supplemented with 10% FBS was used to imitate host environment (Figure 1). Based on preliminary tests, we observed that all concentrations of NE could not stimulate growth of AH196 in serum‐SAPI medium when initial inoculum densities were 10^3^−10^5^ CFU/ml (data not shown). There were no significant differences in OD_600_ among the groups with 0, 12.5, 25, and 50 μM NE additions. When compared to control cultures, the maximum cell density of *Aeromonas hydrophila* AH196 were 1.31‐, 1.27‐, 1.04‐, 1.01‐, and 1.02‐fold higher in 200, 100, 50, 25, and 12.5 μM of NE added serum‐SAPI medium, respectively (at 36, 36, 72, 72, and 72 hr, respectively). Moreover, addition of 100 and 200 μM NE considerably enhanced AH196 growth after 18 hr (*p *<* *0.05). In the second trial experiments, the addition of 100 μM NE significantly stimulated the growth of different *A. hydrophila* isolates AH33, AH189, AH301, and NJ‐35 from cyprinid fish (*p *<* *0.01), and almost doubled the growth stimulation effect of *A. hydrophila* NJ‐35 when compared to control group (Figure 2).

### 3.2 Virulence‐associated genes expression

**Figure 3:**
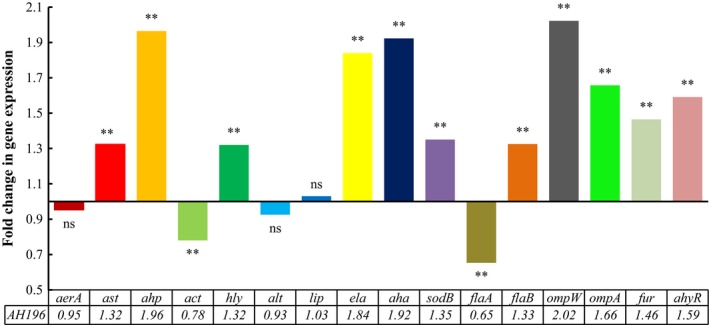
Fold change in the virulence‐associated gene expression profiles of *Aeromonas hydrophila* AH196 after treatment with 100 μM norepinephrine. Virulence‐associated gene expression levels of *A. hydrophila* AH196 were analyzed by qRT‐PCR and normalized to the reference gene *rpoB*. Asterisks indicate a significant difference when compared to untreated *A. hydrophila* (**p < 0.01; *ns*:* p* > 0.05)

Variation in gene expression of *A. hydrophila* AH196 with and without NE addition is shown in Figure 3. NE addition resulted in significantly upregulated expression of *ahp* (1.96‐fold), *ela* (1.84‐fold), *aha* (1.92‐fold), *ompW* (2.02‐fold), *ompA* (1.66‐fold), *fur* (1.46‐fold), *ahyR* (1.59‐fold), *ast* (1.32‐fold), *hly* (1.32‐fold), *sodB* (1.35‐fold), and *flaB* (1.33‐fold) (*p *<* *0.01). In contrast, the addition of NE resulted in markedly downregulated expression of *act* (0.78‐fold) and *flaA* (0.65‐fold) (*p *<* *0.01). There was no statistical significance of the expression of *aerA* (0.95‐fold), *alt* (0.93‐fold), and *lip* (1.03‐fold) after NE addition (*p *>* *0.05).

### 3.3 Protease activity, lipase activity, hemolysis, and swimming motility

**Figure 4:**
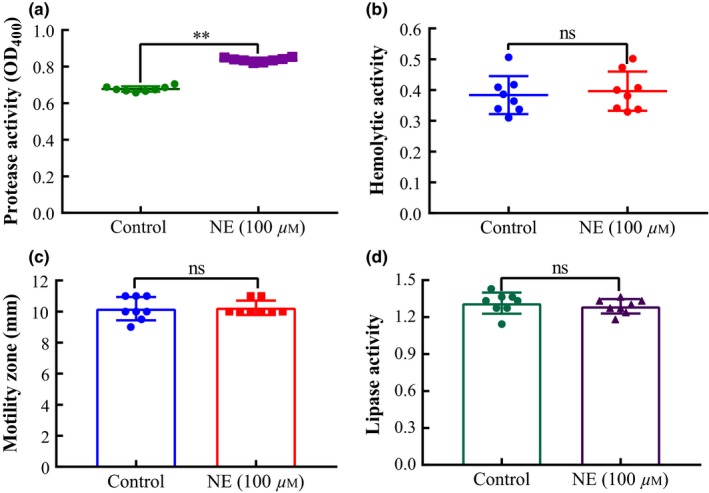
Effect of norepinephrine on protease activity, lipase activity, hemolysis, and swimming motility of *Aeromonas hydrophila* AH196. An initial AH196 density of 10^2^ CFU/ml was cultured to logarithmic growth in the absence or presence of 100 μM norepinephrine (NE), washed twice, and adjusted to equivalent cell densities (OD_600_ = 0.6) in order to determine (a) protease activity via azocasein assays, (b) hemolysis via spectrophotometry, (c) swimming motility on soft serum‐SAPI agar supplemented with 100 μM NE, and (d) lipase activity on serum‐SAPI agar containing 1% Tween 80 and 100 μM NE. **p < 0.01; *ns*: no statistical significance (*p* > 0.05)

The protease activity, lipase activity, hemolysis, and swimming motility of *Aeromonas hydrophila* AH196 were shown in Figure 4. Bacterial cell populations in the NE treatment group showed an observable enhancement in protease activity (Figure 4a; *p *<* *0.01), while significant differences in lipase activity, hemolysis, and motility were not observed when compared to untreated groups (Figure 4b–d; *p *>* *0.05).

### 3.4 Virulence enhancement of *Aeromonas hydrophila* by NE in vivo

**Figure 5:**
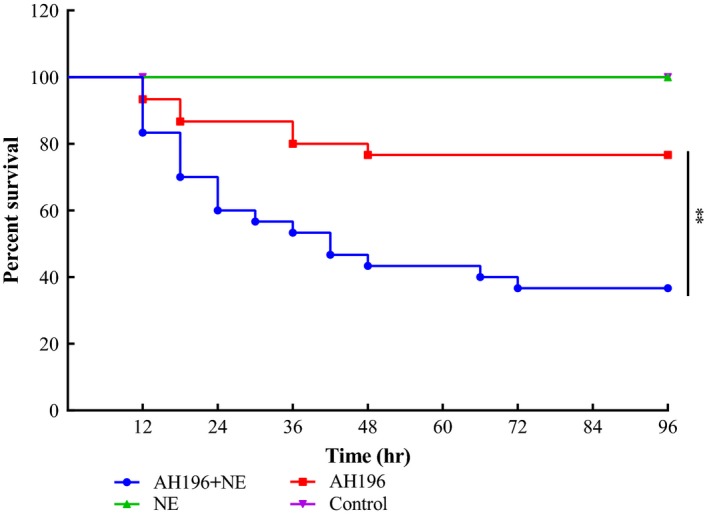
Crucian carp survival with norepinephrine (NE) treatment after *Aeromonas hydrophila* AH196 infection. Crucian carp were inoculated intraperotineally 100 μM norepinephrine or equivoluminal vehicle solvent at 4 hr post infection with 2 × 10^5^ CFU of AH196, and other two groups were separately administered corresponding volumes of norepinephrine and normal saline in order to assess the effects of NE on AH196‐induced mortality (**p < 0.01)

We performed artificial challenge tests and concomitant changes of NE levels in crucian carp in order to assess whether NE can affect *A. hydrophila* AH196 infection and virulence in vivo. Survival data for fish within 96 hr for the four groups (AH196 + NE, AH196, NE, and control) are shown in Figure 5. No fish death was observed in the NE and control groups. In contrast, fish injected with *A. hydrophila* AH196 and saline had a 0.23 ± 0.06 accumulated mortality rate (77% survival). The injection of NE following the infection of *A. hydrophila* AH196 resulted in marked increases in fish mortality rate reaching 0.63 ± 0.15 (37% survival) when compared to other groups (*p *<* *0.01). The moribund fish presented hemorrhagic septicemia symptoms, and bacteria that were isolated from dying fish organs (liver, spleen, and kidney) were identified as *A. hydrophila* AH196.

## 4 DISCUSSION

The addition of NE at 100 and 200 μM markedly accelerated the growth of *Aeromonas hydrophila* AH196 in 36–72 hr (Figure 1), and similar results were observed in other strains (NJ35, AH33, AH189, AH301) that were tested with 100 μM NE treatment at 36 hr (Figure 2). The stimulation of growth by NE is consistent with what has been observed in other bacterial pathogens including *Streptococcus pneumoniae* (Gonzales, Castillo‐Rojas, Castillo‐Rodal, Tuomanen, & López‐Vidal, 2013), *Vibrio harveyi* (Yang et al., 2014), and *Pseudomonas aeruginosa* (Lyte & Ernst, 1992). Under lower NE concentration (12.5, 25, and 50 μM), no significant growth differences were observed in *A. hydrophila* AH196. However, this result was in contrast to previous reports that 10 μM NE could induce log‐fold changes in *A. hydrophila* growth (Dong et al., 2016; Kinney et al., 1999). This difference may be attributed to the variation of experimental conditions including transferrin levels, bacterial strains, and inoculum densities in different studies (O'Donnell et al., 2006). The medium used is crucial to investigate the effect of NE to the bacterial growth or virulence. Most researches mimicked the host iron‐limited condition with serum supplement, in which the iron was sequestered by transferrin. Both adult bovine serum (ABS) and FBS are commonly used medium supplements, and contain bacteriostatic constituents, such as transferrin, complement, and antibodies. However, bovine serum contains essential nutrients for cell growth and its composition and content are often different following the change of the gender, age, physiological condition, and nutritional condition of the blood donors. Based on preliminary tests, we found that NE significantly enhanced the growth of *Aeromonas hydrophilia* AH196 in the medium with ABS and FBS, and a higher growth stimulation of *Aeromonas hydrophilia* AH196 was observed in serum‐SAPI medium containing FBS rather than that of ABS (data not shown). The previous studies also have chosen serum‐SAPI medium supplemented FBS as a culture medium to assess the effect of NE on the growth of *Vibrio cholerae* (Halang et al., 2015), *Aeromonas hydrophilia* (Dong et al., 2016), *Campylobacter jejuni* (Xu et al., 2015), and *Vibrio parahaemolyticus* (Nakano, Takahashi, Sakai, & Nakaya, 2007).

Iron is an indispensable trace element for bacterial growth, proliferation, and virulence. In vertebrates, iron is sequestered by transferrin, a high‐affinity iron‐binding protein in serum, difficult to access by invading pathogenic bacteria. The underlying mechanism for how NE enhances the pathogenic bacteria under iron‐restricted environment has attracted much attentions. It was considered that the catecholamine reduces the ferric iron‐binding affinity of transferrin, which were responsible for the bacteriostatic nature of serum and mucosal secretions (Freestone, Sandrini, Haigh, & Lyte, 2008; Freestone et al., 2007; Sandrini et al., 2014). Recently, Dong et al. (2016) reported that *A. hydrophila* growth stimulation by NE required the TonB2 energy transduction system instead of the amonabactin siderophore, which implies that bacteria contain stress hormone‐related iron acquisition systems.

The pathogenesis of *A. hydrophila* is multifactorial, and characterized by the involvement of a number of virulence factors, such as adhesins (Fang, Ge, & Sin, 2004), outer membrane proteins (omps; Confer & Ayalew, 2012), aerolysins (Howard, Garland, Green, & Buckley, 1987), hemolysins (Asao, Kinoshita, Kozaki, Uemura, & Sakaguchi, 1984), enterotoxins (Chopra, Houston, Peterson, & Jin, 1993; Sha et al., 2005), serine protease (Cascón, Fregeneda, et al., 2000; Méndez et al., 2012), and elastase (Cascón, Yugueros, et al., 2000). Further, *ahyR* encodes a LuxR‐type quorum sensing regulator that regulates the expression of virulence factors in *A. hydrophila* (Kirke, Swift, Lynch, & Williams, 2004; Swift et al., 1997, 1999). Additionally, the iron‐responsive ferric uptake regulator (*fur*) also plays a significant role in iron homeostasis and pathogenesis of *A. hydrophila* (Carpenter, Whitmire, & Merrell, 2009). Adhesion in the host is an important primary step of the infection procedure of pathogenic bacteria. In the present study, the relative expression of *aha*,* ompW,* and *ompA* genes increased significantly in the presence of NE. The protein products of *aha*,* ompW,* and *ompA* gene are crucial adherence and pathogenic factors, located in the outer cell layer, and are involved in maintaining cytoskeletal structure, biofilm formation, transport of nutrient substances, and resistance to host immune defenses (Khushiramani et al., 2012; Maiti, Shetty, Shekar, Karunasagar, & Karunasagar, 2012). The result in this report suggested that NE enhanced the adhering capacity of *A. hydrophila* and accelerated the development of infectious disease, and was consistent with observations by Chen, Lyte, Stevens, Vulchanova, and Brown (2006) that NE stimulated the upregulated expression of the intimin‐encoding gene *eae* in *Escherichia coli* O157:H7. Our results also showed that NE effectively promoted the expression of *flaB* (structural polar flagellin gene), but simultaneously suppressed the expression of polar flagellin structural gene, *flaA* of *A. hydrophila*. Intriguingly, our swimming assay results suggested that NE does not significantly affected the motor ability of *Aeromonas hydrophila*. Combined with the above results, we speculated that the changes in motility might be the consequence of interactive effects of flagellar motility‐related genes. Lateral flagella (laf, another type of flagella in *A. hydrophila*) is responsible for the motility, adherence, and biofilm formation when bacteria grow over viscous environment or surface (Beaz‐Hidalgo & Figueras, 2013; Kirov et al., 2002). Yang et al. (2014) reported that NE notably increased the swimming motility and the expression of polar flagella structural and regulation genes of *Vibrio harveyi*, meanwhile NE upregulated the gene expression of both lateral flagellar flagellin and regulator for threefold, which provided an insight into the effect of NE on bacterial motility mechanisms and pathogenic processes. Worthy to note, the swimming motility in the study was detected using LB35 plate containing 0.3% agar. The majority of *A. hydrophila* strains produce two types of extracellular proteases: a serine protease with caseinolytic activity encoded by the *ahp* gene, and an elastase with both caseinolytic and elastolytic activity encoded by the *ela* gene (Cascón, Fregeneda, et al., 2000; Rivero, Anguita, Mateos, Paniagua, & Naharro, 1991). Both proteases could break down the structure of host cells and tissues, thereby supplying nutrient elements for bacterial growth and propagation, in addition to damaging macrophages (Ascencio & Wadström, 1991). Indeed, NE was effective to promote proteinase activity and alter the expression of *ahp* and *ela* of *A. hydrophila*, which suggested that NE facilitated the infection process and virulence of *A. hydrophila*. The theromstable cytotonic enterotoxin (ast) and hemolysin (hly) are vital exotoxins of *A. hydrophila,* and can promote the hemolysis, cytotoxicity, and enterotoxigenesis (Chopra et al., 1993). Our results also indicated that NE enhanced *ast* and *hly* gene expression of *A. hydrophila*.

*Fur*, an predominant iron‐regulating factor in Gram‐negative bacteria, regulates iron metabolism‐related genes and cellular processes by sensing iron availability in the surrounding environment, such as acid resistance, oxidative and nitrosative stress, chemotaxis, and the expression of virulence factors (Escolar, Pérez‐martín, & De Lorenzo, 1999; Salvail & Massé, 2012). Our results indicated that NE considerably upregulated *fur* and *sodB* gene expression in *A. hydrophila*. To maintain intracellular iron homeostasis, fur activity is activated in iron‐rich environments, while the repression of fur activity is alleviated in low‐iron conditions, which then promotes the synthesis of siderophores to uptake iron (Porcheron & Dozois, 2015). Based on our results, overexpression of *fur* is a reflection of high ferric levels in bacteria. Meanwhile, activation of *fur* inhibits the synthesis of the siderophores. This supports the hypothesis that there are several mechanisms for iron acquisition in *A. hydrophila*. Several transcriptional analyses studies have demonstrated that *sod* was positively regulated by *fur* (Holmes et al., 2005; Oglesby, Murphy, Iyer, & Payne, 2005). Hydroxyl radicals may be produced by fenton chemistry reactions that then result in oxidative stress during iron metabolism (Touati, Jacques, Tardat, Bouchard, & Despied, 1995). Miura, Muraoka, Fujimoto, and Zhao (2000) showed that DNA damage could be induced by catecholamine hormones in the presence of iron. Therefore, the upregulation of *sodB* could result in catalytic conversion of superoxide radicals, thereby promote tolerance to the extremely toxic and oxidative compounds and ultimately enhance *A. hydrophila* viability. This explanation agrees well with previous research that the effect of NE on *sodB* gene expression (Graziano et al., 2014). Sha, Lu, and Chopra (2001) showed that the repression of *act* at the transcriptional level was relieved in *fur* isogenic mutants. Conversely, the upregulated *fur* could repress *act* gene expression, which may explain the downregulation of *act* in NE‐exposed *A. hydrophila*.

*ahyR*, homolog of *LuxR* of *Vibrio fischeri* quorum sensing system, which can coordinate gene expression via sensing the accumulation of signal molecules secreted by *A. hydrophila* (Defoirdt, Boon, Bossier, & Verstraete, 2004; Suga & Smith, 2003). The *ahyR*/*LuxR* could positively regulate the virulence factors expression, serine protease (Rui, Liu, Ma, Wang, & Zhang, 2008), and caseinase activity (Natrah et al., 2011). Here, NE‐induced *ahyR* gene expression and caseinase activity in *A. hydrophila* indicated that NE might be involved in *ahyR*‐mediated expression of virulence factors.

*A. hydrophila* is a well‐acknowledged opportunistic pathogen, and widely occurs in aquaculture environment and the gastrointestine of healthy fish. Fish stress caused by handling, temperature change, low dissolved oxygen and other factors can markedly increase the infection and disease outbreak caused by *A. hydrophila* (Dror et al., 2006; Peters, Faisal, Lang, & Ahmed, 1988). It seems like that *A. hydrophila* could sense and respond to the stress hormone of fish host. Therefore, in this report authors used an in vivo challenge model by injecting pathogenic bacteria *A. hydrophila* and exogenous stress hormone NE to confirm the affect of stress hormone on pathogenic bacteria infection. The LD_50_ of *A. hydrophila* AH196 in crucian carp challenged with intraperitoneal injection was 3.7 × 10^6^ CFU/ml. To acquire the strongest possible virulence enhancement by NE, a lower concentration (1 × 10^6^ CFU/ml) of bacterial inocula was employed in our study. Our findings showed that NE increased the proliferation and expression of virulence‐related genes in *A. hydrophila*, and the death rate of crucian carp. In vivo challenge tests in crucian carp agreed well with previous reports that virulence enhancement associated with NE exposure in *Vibrio campbellii* (Pande, Suong, Bossier, & Defoirdt, 2014), *Vibrio harveyi* (Yang et al., 2014), and *Vibrio parahaemolyticus* (Suong et al., 2017). Hence, the exogenous stress hormone NE can enhance the virulence and pathogenicity of *A. hydrophila* in fish host. However, further studies are needed to reveal how stress hormone NE enhances the growth and virulence of *A. hydrophila*.

## CONFLICT OF INTEREST

The authors declare that the research was conducted in the absence of any commercial or financial relationships that could be construed as a potential conflict of interest.
